# Supplementary material for: Primary Sclerosing Cholangitis: Burden of Disease and Mortality Using Data from the National Rare Diseases Registry in Italy
Source: Int J Environ Res Public Health. 2020 Apr 29;17(9):3095. doi: 10.3390/ijerph17093095 (PMC7246900; doi:10.3390/ijerph17093095)
Supplement: Supplementary file 1 [file ijerph-17-03095-s001.pdf]

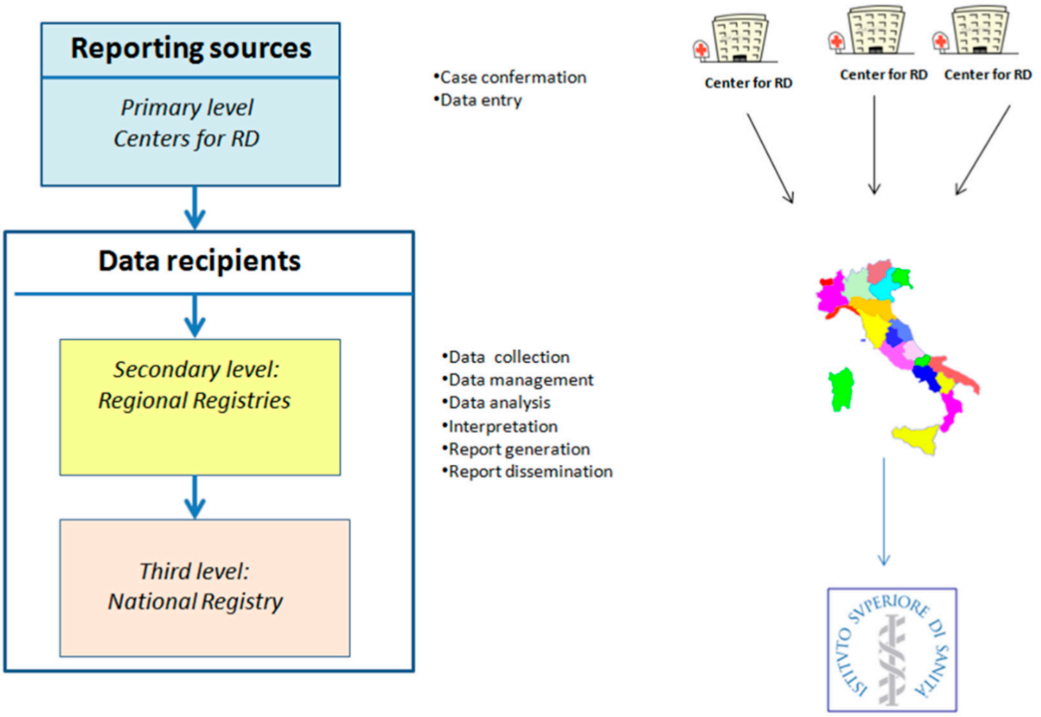

Figure S1. Data flow within the italian National Rare Diseases Registry.

Table S1. Regional distribution of incident cases with primary sclerosing cholangitis and incidence rate per 100,000 individuals during 2012–2014 in Italy.

Italian Region	Cases(2012)	Resident population (Italy 2012)	Incidence	Cases 2013	Resident population (Italy 2013)	Incidence	Cases (2014)	Resident population (Italy 2014)	Incidence	Total cases (2012-2014)	Population (2012-2014)
**Abruzzo**	3	1,312,507	0.23	0	1,333,939	0.00	0	1,331,574	0.00	3	3,978,020
**Basilicata**	0	576,194	0.00	1	578,391	0.17	0	576,619	0.00	1	1,731,204
**Calabria**	1	1,958,238	0.05	0	1,980,533	0.00	0	1,976,631	0.00	1	5,915,402
**Campania**	0	5,769,750	0.00	3	5,869,965	0.05	1	5,861,529	0.02	4	17,501,244
**Emilia-Romagna**	13	4,377,487	0.30	10	4,446,354	0.22	11	4,450,508	0.25	34	13,274,349
**Friuli-Venezia Giulia**	0	1,221,860	0.00	10	1,229,363	0.81	1	1,227,122	0.08	11	3,678,345
**Lazio**	3	5,557,276	0.05	0	5,870,451	0.00	1	5,892,425	0.02	4	17,320,152
**Liguria**	1	1,565,127	0.06	4	1,591,939	0.25	2	1,583,263	0.13	7	4,740,329
**Lombardia**	4	9,794,525	0.04	2	9,973,397	0.02	5	10,002,615	0.05	11	29,770,537
**Marche**	4	1,545,155	0.26	0	1,553,138	0.00	1	1,550,796	0.06	5	4,649,089
**PA**	3	509,626	0.59	0	515,714	0.00	3	518,518	0.58	6	1,543,858

**Bolzano**										
**Pa Trento**	1	530,308	0.19	0	536,237	0.00	0	537,416	0.00	1	1,603,961
**Piemonte**	10	4,374,052	0.23	3	4,436,798	0.07	6	4,424,467	0.14	19	13,235,317
**Puglia**	1	4,050,803	0.02	24	4,090,266	0.59	6	4,090,105	0.15	31	12,231,174
**Sardegna**	1	1,640,379	0.06	0	1,663,859	0.00	0	1,663,286	0.00	1	4,967,524
**Sicilia**	0	4,999,932	0.00	1	5,094,937	0.02	0	5,092,080	0.00	1	15,186,949
**Toscana**	3	3,692,828	0.08	4	3,750,511	0.11	1	3,752,654	0.03	8	11,195,993
**Umbria**	1	886,239	0.11	1	896,742	0.11	6	894,762	0.67	8	2,677,743
**Valle d'Aosta**	1	127,844	0.78	0	128,591	0.00	0	128,298	0.00	1	384,733
**Veneto**	6	4,881,756	0.12	8	4,926,818	0.16	15	4,927,596	0.30	29	14,736,170

Table S2. Characteristics of 25 individuals with primary sclerosing cholangitis who had died.

Case	Age at death (years)	ICD-10 code	Cause of underlying death
1	45	C18.9	Colon, unspecified
2	49	C18.9	Colon, unspecified
3	50	C22.1	Intrahepatic bile duct carcinoma
4	53	C22.1	Intrahepatic bile duct carcinoma
5	67	C23	Malignant neoplasm of gallbladder
6	35	C85.9	Non-Hodgkinlymphoma, unspecified
7	70	D47.1	Chronic myeloproliferative disease
8	46	D84.9	Immunodeficiency, unspecified
9	57	E14.5	Unspecified diabetes mellitus with peripheral circulatory complications
10	60	H44.0	Purulentendophthalmitis
11	79	I61.9	Intracerebralhaemorrhage, multiple localized
12	43	I67.8	Other specified cerebrovascular diseases
13	76	K74.6	Other and unspecified cirrhosis of liver
14	45	K83.0	Cholangitis
15	47	K83.0	Cholangitis
16	56	K83.0	Cholangitis
17	45	K83.0	Cholangitis
18	70	K83.0	Cholangitis
19	72	K83.0	Cholangitis
20	65	K83.0	Cholangitis
21	75	K83.0	Cholangitis
22	62	N18.9	Chronickidney disease, unspecified
23	86	N39.0	Urinary tract infection, site not specified
24	71	S72.9	Fracture of femur, part unspecified
